# Survival and Infectivity of the Insect-Parasitic Nematode *Heterorhabditis bacteriophora* Poinar in Solutions Containing Four Different Turfgrass Soil Surfactants

**DOI:** 10.3390/insects4010001

**Published:** 2012-12-20

**Authors:** Terri L. Hoctor, Timothy J. Gibb, Cale A. Bigelow, Douglas S. Richmond

**Affiliations:** 1 Department of Entomology, Purdue University, West Lafayette, IN 47907, USA; E-Mails: gibb@purdue.edu (T.J.G.); drichmond@purdue.edu (D.S.R.); 2 Department of Agronomy, Purdue University, West Lafayette, IN 47907, USA; E-Mail: cbigelo1@purdue.edu

**Keywords:** *Heterorhabditis bacteriophora*, surfactant, survival, infectivity, tank-mixing, integrated pest management

## Abstract

This laboratory study examined viability and infectivity of the entomopathogenic nematode (EPN) *Heterorhabditis bacteriophora* Poinar in solutions containing four different turfgrass soil surfactants: *Revolution* (Aquatrols Corp., Paulsboro, NJ), *Aqueduct* (Aquatrols Corp., Paulsboro, NJ), *Cascade Plus* (Precision Laboratories Inc., Waukegan, IL) and *OARS* (Aqua-Aid Inc., Rocky Mount, NC). Infective juvenile *H. bacteriophora* were added to solutions containing each of the four surfactants, and nematode viability and infectivity were monitored over time. In one of two trials, nematode survival in solutions containing the surfactants *Aqueduct* and *Cascade Plus* was consistently higher compared to the water control and solutions containing *Revolution* or *OARS*. Surfactants had no significant influence on nematode infectivity in either trial. Findings indicate that most of the common turfgrass soil surfactants examined should be compatible with EPNs and that some may potentially enhance nematode survival. Results also imply that tank-mixing of EPNs with most turfgrass soil surfactants should not pose a significant risk to the nematodes. The influence of soil surfactants on EPN performance remains to be examined in the field.

## 1. Introduction

### 1.1. EPNs used for Biological Control in Turfgrass

In order for Entomopathogenic nematodes (EPNs) to be an effective form of biological control, appropriate matching of nematode host finding behaviors with the behavior and habitat of the target pest is essential. Entomopathogenic nematodes (EPNs) are microscopic roundworms that parasitize and kill many insect pest species. These nematodes penetrate their insect host through the mouth, anus or spiracles, or by passing directly through the insect cuticle. Once inside, EPNs release a species-specific bacterial symbiont that multiplies exponentially within the insect’s body, killing the insect within 24 to 48 hours [1]. Two genera of nematodes within the order *Rhabditida*, the Steinernematids and Heterorhabditids, have both had some success has biocontrol agents [2]. However, insect pests must be managed with a nematode species that employs the appropriate host-seeking strategy [3,4]. For example, the EPN *Steinernema carpocapsae* Wieser employs an ambush strategy that effectively controls insects such as caterpillars and chinch bugs that are active at or near the soil surface. In contrast, *Heterorhabditis bacteriophora* Poinar works beneath the soil surface employing a cruising strategy to target less active soil dwelling pests such as white grubs which are generally considered the most destructive pests of turfgrass [5,6,7]. 

### 1.2. EPN Survival in Turfgrass

When used against an appropriate insect host, entomopathogenic nematodes (EPNs) represent one of the relatively few promising biological alternatives for managing insect pests of turfgrass. However, application techniques and ultimately field survival pose significant challenges to more widespread adoption of these biological controls. Soil moisture is one of the most important factors influencing the survival, persistence and infectivity of EPNs in the field. EPNs require a film of water in order to move and find suitable insect hosts [8]. In areas of extremely low soil moisture, nematodes cannot successfully locate and penetrate the host before desiccation leads to loss of movement and eventual death [9]. Although infective juvenile nematodes (IJs) are capable of surviving in dry soils for a considerable period of time [10] by remaining inside a host cadaver [11], post application mortality of IJs can be substantial. Depending on species, EPN mortality may reach 40%–80% due to UV exposure and desiccation four hours following application. Desiccation in conjunction with predators and pathogens can continue to reduce EPN viability in the soil by an additional 5–10% each day thereafter [12]. Therefore, it is very important to be able to move the applied EPNs off the leaf blade and into the soil. Management inputs that enhance soil moisture levels, reduce fluctuations in soil moisture, or protect EPNs from desiccation during the first few critical hours following application could significantly enhance EPN survival and efficacy. To date, relatively few studies have examined the potential for turfgrass management inputs to enhance the survival and performance of EPNs.

### 1.3. Soil Surfactants

Water repellency or hydrophobicity is becoming a widely reported problem in agricultural and turfgrass soils [13]. Soil surfactants, wetting agents or soil penetrants are terms used to describe surface active materials that lower the interfacial tension between a hydrophilic and non-wettable hydrophobic phase. Various commercial products have been used for the past six decades in turfgrass management to alleviate the negative effects of soil water repellency. In general, surfactants work to improve the way water moves across or through the soil profile and increase water efficiency by managing water repellency and distribution/uniformity [14]. Some soil surfactants act as detergents that solubilize soil contaminants [15], allowing water to move through the soil profile. Recently, research evaluating the success of different chemistries of commercial surfactants indicated that the efficacy of these products varied by measured soil depth, but was most pronounced at 2.5 cm or less [16]. In addition to soil water repellency, common cool-season spreading lawn grasses such as Kentucky bluegrass (*Poa pratensis*) often generate a potentially hydrophobic thatch layer at the soil surface. The combination of a hydrophobic soil and thatch layer has the potential to severely limit EPN survival and efficacy. Thus, the utilization of a surfactant may aid in EPN viability.

Recent work combining nematode application with surfactants has resulted in increased nematode efficacy against codling moth in apple storage facilities [17,18] and pecan weevil in greenhouse studies [19]. Similarly, the compatibility of EPNs with various insecticides [20] and herbicides [21] has been previously examined. However, the compatibility of EPNs with soil surfactants designed specifically for use in turfgrass environments has not been explored. As a result, the potential for enhancing nematode efficacy by pre-treating the soil/thatch with a surfactant, or tank-mixing such products with EPNs, remains unclear. Simultaneous nematode/surfactant applications or the development of nematode/surfactant/ application programs could improve the cost-effectiveness of EPNs and enhance their persistence in the soil environment making them a more viable option for biological control in turfgrass environments.

The objective of this research was to examine the compatibility of several commercially available surfactants with the EPN, *Heterorhabditis bacteriophora* Poinar, under laboratory conditions. Nematode persistence was monitored over time in solutions containing four different turfgrass surfactants and the infectivity of EPNs taken from those solutions was examined using larvae of the greater wax moth, *Galleria mellonella* Linnaeus (waxworms).

## 2. Results and Discussion

### 2.1. EPN Survival in Surfactant Solutions

**Figure 1:**
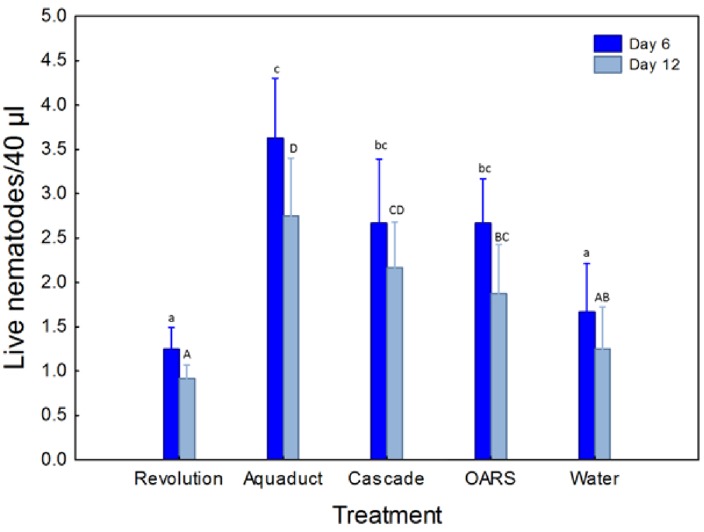
Mean (±SE) number of live infective juvenile *Heterorhabditis bacteriophora* Poinar per 40 µL sample taken from Petri dishes containing one of four different surfactant solutions or water over the course of 12 days. Means with the same letter are not significantly different at α = 0.05. Comparisons at 6 days described by lower-case letters and comparisons at 12 days described by upper-case letters.

Nematode survival varied significantly among the surfactant treatments (F = 2.97; df = 8.66; P = 0.007) (Figure 1). Six days after being added to the experimental solutions, EPN survival in *Aqueduct®, Cascade Plus*™, and *OARS®* solutions was significantly higher compared to the water control (P < 0.05). Survival in the *Revolution®* solution on day six was not significantly different from the water control (P > 0.05). At 12 days, the *Aqueduct®* and *Cascade Plus*™ solutions resulted in significantly higher EPN survival than the water control. Although nematode survival in the *Revolution®* solution was significantly lower than the *OARS®* solution (P > 0.05), neither of these solutions resulted in nematode survival that was significantly different from the water control (P < 0.05). 

### 2.2. EPN Infectivity

Nematode infectivity, as measured by the percentage of infected waxworms (x/4), did not vary significantly among the experimental solutions (F = 177.1; df = 4, 24; P = 0.68) with all surfactant solution yielding infection rates similar to the water control. Across all treatments, the mean percentage of waxworm larvae infected by EPNs taken from the Petri dishes after one week in solution was 9.2 ± 4.5%.

## 3. Experimental Section

### 3.1. Soil Surfactant/Nematode Solutions

Four commercially available surfactants were used in this experiment: *Revolution* (Aquatrols Corporation, Paulsboro, New Jersey), *Aqueduct*® (Aquatrols Corporation, Paulsboro, New Jersey), *Cascade Plus*™ (Precision Laboratories, Waukegan, Illinois) and *OARS* (Aqua-Aid, Rocky Mount, North Carolina). Stock solutions of these products were prepared using filtered spring water (Magnetic Springs, Columbus, OH) according to label recommendations (Table 1). Fresh solutions were prepared just prior to use in the experiment.

Infective juvenile (IJ) *H. bacteriophora* (Nemasys® G, Becker Underwood, Ames, IA) were cycled through waxworms to ensure that fresh, healthy IJs were available for experimentation. IJs were added to Petri dishes (14 cm diameter) at the standard field rate of 2.5 billion/ha (3800 nematodes/Petri dish) in 2.7 mL of water using a micro-pipetter. Prior to adding the nematodes to the experimental solutions, nematodes were stored in water at 10 °C. These nematodes were subsampled and the number of viable nematodes were counted in three 50 µL samples. The mean number of live nematodes for a given volume was determined and was used to evenly add nematodes to the Petri dishes. One of the four surfactant solutions or filtered water (57.3 mL) was then added to each Petri dish for a total volume of 60 mL/dish. The total volume of the solutions was held constant by adding from stock surfactant solutions every three days or as needed. Petri dishes containing the nematodes/surfactant solutions were held on the laboratory bench at room temperature (20 °C). Each treatment was replicated a total of ten times in two experimental blocks of five replicates each and all data were pooled prior to analysis. 

### 3.2. EPN Survival in Surfactant Solutions

To monitor nematode viability, three subsamples of the nematode solution (40 µL) were taken from each Petri dish at 6 and 12 days after the nematodes were initially placed into the various experimental solutions using a micro-pipetter. The number of live nematodes in each subsample was determined by dispensing the entire subsample on a glass microscope slide and observing the sample under a stereo microscope. The number of live nematodes per subsample was recorded and the three subsample mean was reported for analysis. 

### 3.3. EPN Infectivity

One week after the nematode/ surfactant solutions were prepared for the EPN survival study, the, infectivity of the live infective juvenile nematodes in each stock solution was quantified using waxworms. Waxworms were placed individually into the wells of a 24 well-plate, each containing 2.5 g of fine washed silica sand moistened to 20% water by weight. One infective juvenile nematode was taken from each of the four surfactant solutions and the water control and applied in 10 µL to the surface of the sand in each well using a micro-pipetter. Lids were placed on the plates and plates were held at room temperature on the laboratory bench. After five days, waxworms were examined for infection as indicated by characteristic color change [11,22]. Only waxworms that were positively infected by the nematodes were counted and waxworm mortality from other unknown sources was not considered. Each treatment was replicated 6 times, utilizing 6 of the 10 replicated Petri dishes from the EPN survival experiment (3 from each block). The experiment was conducted in 2 blocks of three replicates each and a total of four waxworms were used for each replicate (n = 24).

### 3.4. Statistical Analysis

Variation in nematode viability was examined over time using multivariate analysis of variance (MANOVA) with the mean number of live nematodes/40 µL at 6 and 12 days after exposure serving as the dependent variable. The independent variable was the surfactant treatment (or water control). Because treatment effects were statistically significant in the MANOVA, univariate results were examined to identify sampling dates on which significant treatment effects were observed. Comparisons between treatments were made on individual sampling dates using Fisher’s LSD test (α = 0.05). Variation in the proportion of waxworms infected by nematodes taken from each experimental solution was examined using univariate analysis of variance with the arcsin square root transformed percentage of infected waxworms serving as the dependent variables. Again, the independent variable was the surfactant treatment (or water control). Treatment means were compared using Fisher’s LSD test (α = 0.05) and all statistical analyses were performed using Statistica 9 (Statsoft Inc., Tulsa, OK, USA). 

**Table 1:** Field application rates for surfactant products used in laboratory assays.

Trade Name	Manufacturer	Dilution
*Revolution*	Aquatrols Corp.	1L product/43L H_2_0/km^2^
*Aqueduct*	Aquatrols Corp.	1L product/16L H_2_0/km^2^
*Cascade Plus*	Precision Laboratories	1L product/32L H_2_0/km^2^
*OARS*	Aqua-Aid Inc.	1L product/43L H_2_0/km^2^

## 4. Conclusions

### 4.1. Summary and Implications

Soil surfactants are routinely used in turfgrass systems to improve soil/thatch wettability and ultimately turf health [13]. Results of the current study indicate that *in situ*ations where tank-mixing nematodes with surfactants is desirable, most surfactants, should pose little risk to EPNs. Furthermore, results suggest that the addition of wetting agents to the soil should not be detrimental to nematode longevity or infectivity and that several of the surfactants studied may actually enhance nematode longevity. In particular, *Aquaduct*® and *Cascade Plus*™, both of which are ethylene oxide/propylene oxide (EO/PO) block copolymer surfactants, resulted in the highest nematode survival. Neither the methyl-capped block copolymer surfactant *Revolution*® nor the surfactant/organic solvent complex *OARS* provided any significant benefit over water alone. These findings indicate that surfactant class/chemistry may be an important determinant of the degree of compatibility with EPNs. While the persistence of EPN populations could potentially be enhanced by the use of certain turfgrass soil surfactants, EPN infectivity does not appear to be significantly influenced. Therefore, by manipulating the soil environment via surfactants it may be possible to reduce infective juvenile EPN mortality thereby increasing nematode efficacy against turfgrass pests; particularly soil insects (e.g., white grubs and billbugs). The post-application population dynamics of EPNs in combination with the use of surfactants should be more closely examined under field conditions. Normally surfactants are applied alone and quickly watered into the soil, not-tank-mixed with other products such as EPNs. The present study demonstrates the potential compatibility of four commercial surfactants with EPNs. 
